# SDC-DeepLabv3+: Lightweight and Precise Localization Algorithm for Safflower-Harvesting Robots

**DOI:** 10.34133/plantphenomics.0194

**Published:** 2024-07-05

**Authors:** Zhenyu Xing, Zhenguo Zhang, Yunze Wang, Peng Xu, Quanfeng Guo, Chao Zeng, Ruimeng Shi

**Affiliations:** ^1^ College of Mechanical and Electrical Engineering, Xinjiang Agricultural University, Urumqi 830052, China.; ^2^ Key Laboratory of Intelligent Equipment and Robotics for Agriculture of Zhejiang Province, Hangzhou 310058, China.; ^3^ Key Laboratory of Xinjiang Intelligent Agricultural Equipment, Urumqi 830052, China.

## Abstract

Harvesting robots had difficulty extracting filament phenotypes for small, numerous filaments, heavy cross-obscuration, and similar phenotypic characteristics with organs. Robots experience difficulty in localizing under near-colored backgrounds and fuzzy contour features. It cannot accurately harvest filaments for robots. Therefore, a method for detecting and locating filament picking points based on an improved DeepLabv3+ algorithm is proposed in this study. A lightweight network structure, ShuffletNetV2, was used to replace the backbone network Xception of the traditional DeepLabv3+. Convolutional branches for 3 different sampling rates were added to extract information on the safflower features under the receptive field. Convolutional block attention was incorporated into feature extraction at the coding and decoding layers to solve the interference problem of the near-color background in the feature-fusion process. Then, using the region of interest of the safflower branch obtained by the improved DeepLabv3+, an algorithm for filament picking-point localization was designed based on barycenter projection. The tests demonstrated that this method was capable of accurately localizing the filament. The mean pixel accuracy and mean intersection over union of the improved DeepLabv3+ were 95.84% and 96.87%, respectively. The detection rate and weights file size required were superior to those of other algorithms. In the localization test, the depth-measurement distance between the depth camera and target safflower filament was 450 to 510 mm, which minimized the visual-localization error. The average localization and picking success rates were 92.50% and 90.83%, respectively. The results show that the proposed localization method offers a viable approach for accurate harvesting localization.

## Introduction

Safflower is a specialty economic crop with a combination of medicinal herbs, dyes, oil, and fodder and is thus a key pillar of our society [1]. The fruit balls of safflowers contain multiple clusters of small, compact, and dense safflower filaments. The entire life cycle is harvested continuously for 3 to 5 crops [2,3]. Filaments that are not immediately harvested from the fruit ball affect subsequent opening, resulting in reduced filament production [4,5]. Current safflower-crop production relies on extensive labor, negatively impacting production and the lives of flower farmers. Agricultural robots have the potential to revolutionize standard practices [6]. Segmenting safflower filaments can effectively reduce field operations and improve filament yield through robotic identification [7,8]. However, the near-color background and contour edge features of the filament harvesting points are blurred owing to environmental disturbances (weather and light), making their location difficult [9,10]. Meanwhile, safflower has a high number of filaments and phenotypic color characteristics similar to those of fruit balls or branches. It is difficult to extract the filament and organ phenotypes because of the serious cross-obscuration between the filaments and the fruit ball organs. Furthermore, it is hard to accurately localize the filaments by phenotypic feature segmentation and reduce the filament damage rate under the premise of maintaining the original structure of filaments. Therefore, according to the semantic information and phenotypic parameters of the morphological structure of filaments, the method of detecting and localizing safflower filament harvesting points is constructed based on the image segmentation algorithm in the complex environment. The harvesting robot can accurately localize and harvest the safflower filaments with high efficiency and low damage. The method can conveniently and accurately extract the morphological structure phenotypic parameters of filaments and organs. It provides effective technical support for safflower phenotype research.

Recently, deep-learning-based flower-classification methods have been applied to various types of flower-detection problems [11–13]. Most used convolutional neural networks containing multilayer stacked structures for flower color, shape, and appearance features to obtain better detection results. Dias et al. [14] proposed an improved end-to-end residual convolutional neural network to enhance color sensitivity. The flowers were accurately identified by applying DEEPLAB+RGR with an effective recall and accuracy higher than 90%. Tian et al. [15] proposed a method for introducing single-shot multiBox detector deep-learning techniques to color detection and recognition. The average accuracy on the flower dataset was 83.64%. Williams et al. [16] used Faster R-CNN for training on a kiwifruit-flower dataset. The average accuracy of the algorithm was 85.3%. Palacios et al. [17] proposed a field Mikania-recognition model based on deep convolutional neural networks (DCNNs). The accuracy and computing time of the algorithm were improved by fusing the local response normalization function of AlexNet with the continuous convolutional structure of VGG-Net. The recognition accuracy was 94.50%. Gong et al. [18] open a new way to precisely segment fruit target with a learning-based reconstruction approach, which extensively extends the practical in situ semantic recognition performance in complex field scenarios.Zhou et al. [19] optimized the model structure of VGGNet and preferred an 8-layer network algorithm for tomato-flower shape feature extraction during different flower periods. The detection accuracy was 84.48%. Xiong et al. [20] recognized and segmented the shape features of litchi flowers that were densely aggregated in complex natural environments by constructing a 34-layer ResNet backbone network. The mean average precision was 87%. Zhao et al. [21] proposed a cascade convolutional neural network-based tomato-bouquet recognition method. To realize the extraction of the bouquet region of tomatoes and accurate recognition of the flowering period in the bouquet, the average detection accuracy reached 82.79%. Although the aforementioned methods address the detection problem in terms of flower features, they are only applicable to cases with obvious differences (color and brightness) between the target and background. Moreover, most studies have only focused on flower detection [22,23]. Furthermore, few studies have developed simultaneous extraction and picking localization.

Several studies have shown that the most important feature of DeepLabV3+ is the introduction of null convolution compared with the latest improved PSPNet [24], SegNet [25], U-Net [26], MDE-UNet [27], CDMS [28], and CondInst [29] algorithms. The sensory field is increased without loss of information. Each convolution output contains a large range of information, which is beneficial for extracting multiscale information [30,31]. DeepLabV3+ achieved superior segmentation results for various targets. Zhu et al. [32] proposed a 2-stage model, LD-DeepLabv3+, with adaptive loss, to address the problems of hard samples and pixel-proportion imbalance in diseased apple-leaf image segmentation. Peng et al. [33] focused on the semantic segmentation of branches to enable robotic litchi-fruit harvesting by clamping/shearing bearing branches. Therefore, to improve the recognition and localization accuracy of safflower-harvesting robots, this study uses the DeepLabV3+ segmentation algorithm, which has a high accuracy and lightweight structure. A method for detecting and localizing filament picking points based on an improved DeepLabv3+ algorithm is proposed. The improved DeepLabv3+ adopts the lightweight network ShuffletNetV2 to replace the backbone network, improves atrous spatial pyramid pooling (ASPP), and incorporates the convolutional block attention module (CBAM), reducing background interference and enhancing target features. An improved DeepLabv3+ algorithm was used to segment the filament, fruit ball, and branches. The line features of the branch and minimum distance constraint from the barycenter of the filament and fruit ball to the line of the branch were searched and solved to locate the picking-point position. The main contributions of the proposed approach are as follows:

1. DeepLabV3+ image segmentation with high accuracy and a lightweight structure is used as the basis for the algorithm to reduce the interference of background regions and contour edges on the filament, fruit ball, and backbone segmentation. A lightweight network, ShuffletNetV2, is used as the backbone network. The input safflower features are operated during channel separation and a channel-blending operation was performed to ensure that the branch safflower-feature information is fully fused. Subsequently, the downsampling unit directly increases the number of network channels and network width to enhance the capability of the network to extract safflower features.

2. According to the geometrical characteristics of the filaments, the dilated depth-separable convolution replaces the original ASPP module convolution. Three branches of convolution at different sampling rates are added to extract information on the safflower features under the receptive field. The detection effect improves after the introduction of the CBAM. The problems of missed and incorrect segmentation are effectively solved using light intensity and crop shading.

3. Combined with obtaining the regions of interest (ROIs) of safflower branches, the line segments are solved using the Hough straight-line detection algorithm. Concurrently, a picking-point localization algorithm is designed to locate filaments based on barycenter projection with respect to safflower features. Recognition accuracy and real-time performance are effectively improved. It is proposed to provide technical support for safflower-harvesting robots to locate and harvest safflowers with high efficiency and minimal damage.

The remainder of this paper is organized as follows. The “Materials and Methods” section explains the relevant materials and methods. The “Localization Test of Filament Picking Point” section describes the localization test of the filament picking point in this study. The “Results” section presents the experimental results. The "Discussion" section presents a discussion of the results and future work.

## Materials and Methods

### Image data acquisition

**Fig. 1.**
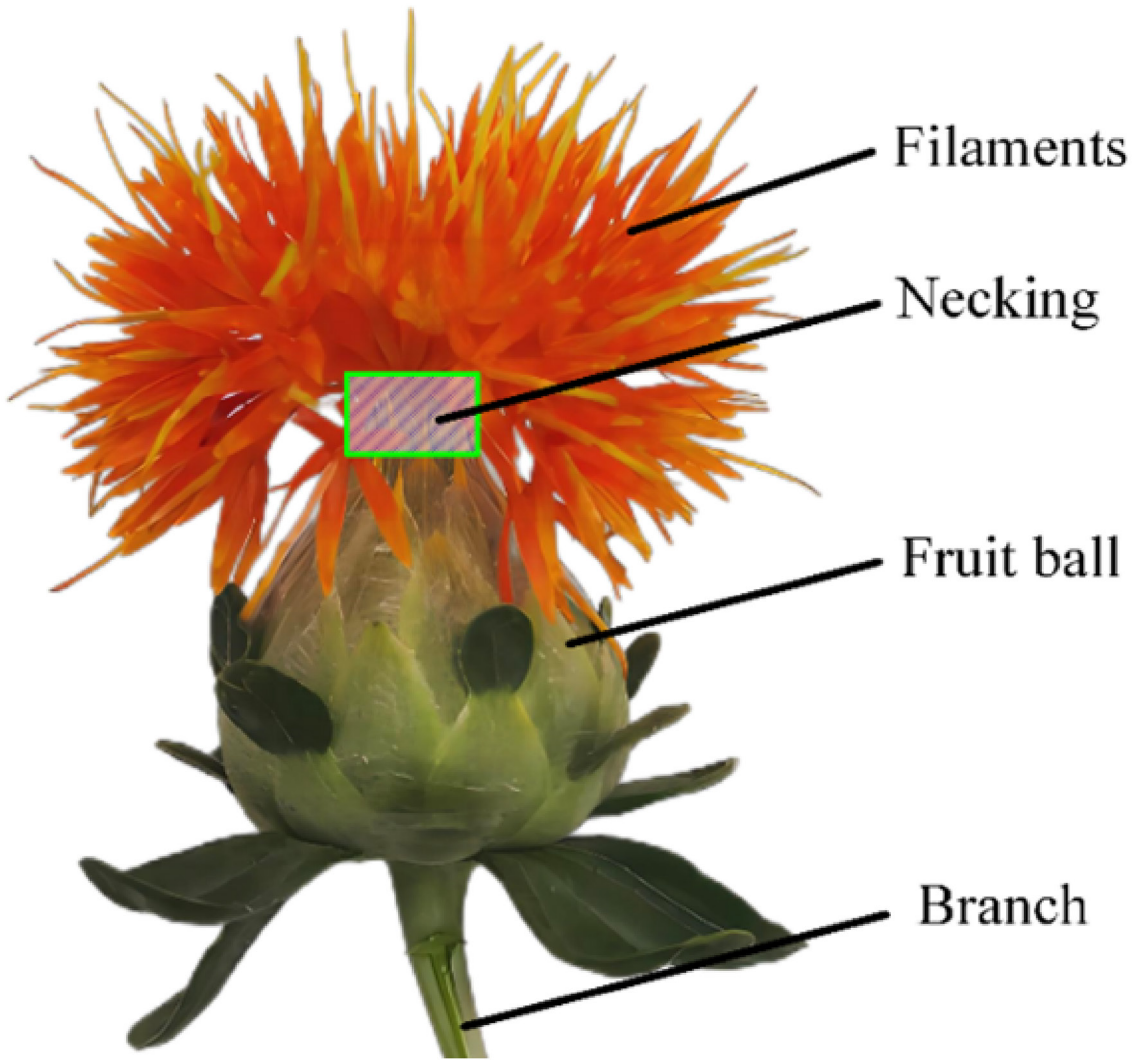
Structure diagram of the opening safflower.

The safflower-image data used in this study were collected in late July 2023 from a safflower planting base in Yili, Xinjiang, China. Safflower grows naturally upward with filaments opening on the fruit ball. The area connecting the filament and fruit ball is the necking (green boxed area in Fig. 1), which is the best harvesting site for the filament picking point. A safflower branch is typically located directly below the filament, fruit ball, and necking, as shown in Fig. 1.

**Fig. 2.**
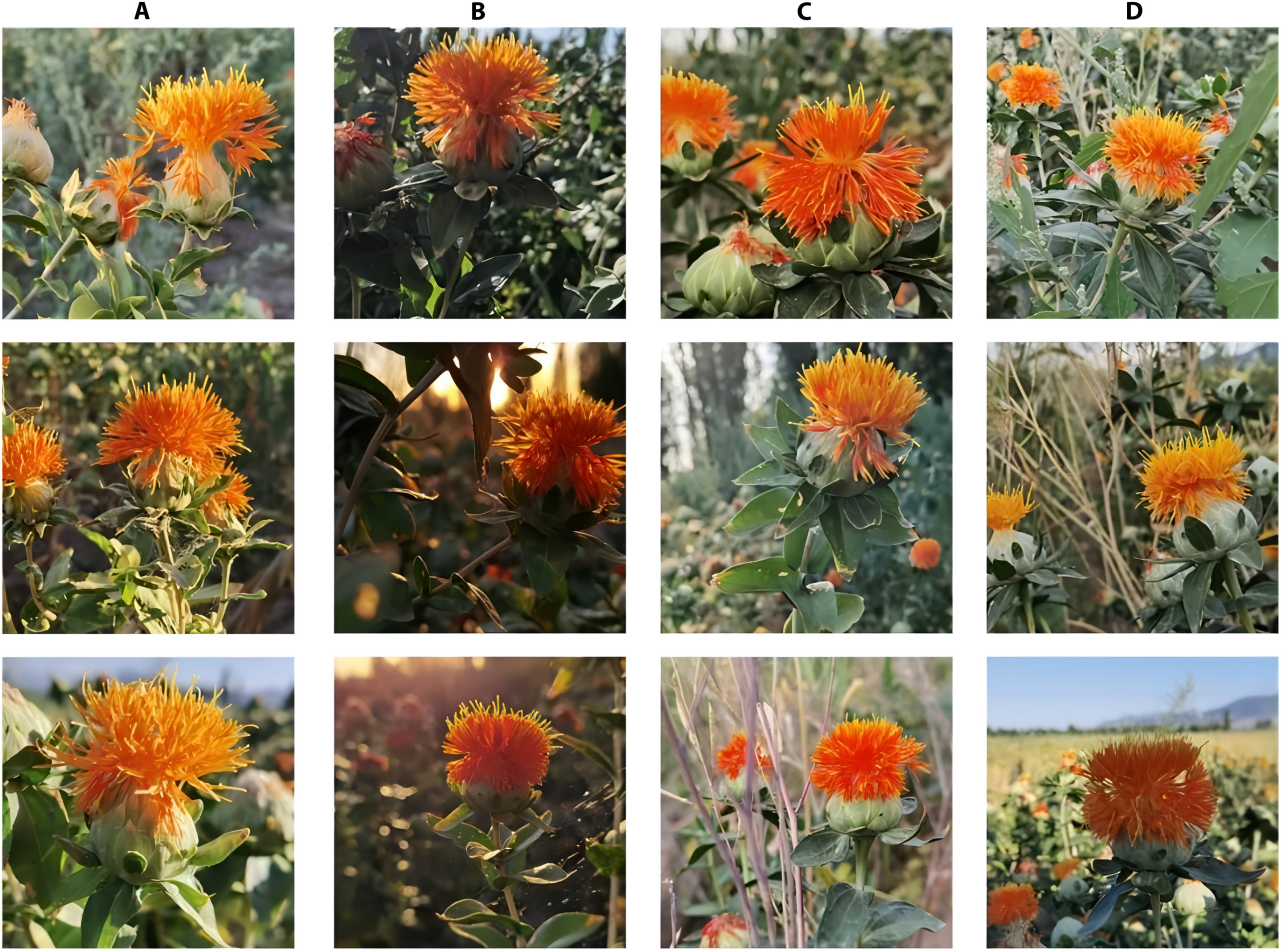
Examples of safflower images. (A) Sunny day with light, (B) sunny day with backlight, (C) overcast day with light, and (D) cloudy day with light.

A D435i depth camera (Intel Corp., USA) was used as the image-acquisition equipment. The image size was 640 × 480 pixels and the format of the acquired images was .png. The acquired images included different weather, light, and shade conditions. A total of 525 safflower-filament images were obtained, as shown in Fig. 2.

### Dataset production

The data were enhanced using geometric (translation and rotation) and color transformations (contrast and brightness) to expand the dataset to 1,050 pictures. The data were divided into training and test sets in a ratio of 8:2, including 840 pictures for the training set and 210 pictures for the validation set. The training set was used to train the deep network model-parameter weights, and the test set was used to evaluate the generalization ability of the final model [34,35]. The filament, fruit ball, and branch were labeled separately using the LabelIme tool. The category information is filament, fruit ball, and branch, which is stored in the .json format.

### DeepLabv3+ algorithm

DeeplabV3+ is a semantic segmentation algorithm based on a DCNN that mainly consists of an encoder [36], decoder [37], DCNN [38], and ASPP [39]. First, the image input to the encoder is fed into the backbone feature-extraction network, Xception. Second, the parallel ASPP module is passed in to incorporate the pooled features at the image level to merge and extract multiscale contextual information. Then, the number of channels is reduced by a 1 × 1 convolution and output to the decoder for 4-fold upsampling. Fusion is performed using shallow features extracted from the DCNN module. Finally, the fused features are passed through a 3 × 3 convolution and 4-fold upsampling to obtain the predicted image, which has the same size as the original image [40].

The high-accuracy DeepLabv3+ algorithm obtains rich image-boundary information and multiscale features. It is suitable for solving problems with no obvious differences and blurred contour-edge features between the target to be segmented and background of small-volume safflower filaments in complex environments [33,41]. However, its network parameters are large, and its prediction speed is slow. It also produces missed and incorrect segmentation problems, resulting in a large deviation in the localization accuracy. Therefore, the detection algorithm must be further optimized.

### Improved DeepLabv3+

**Fig. 3.**
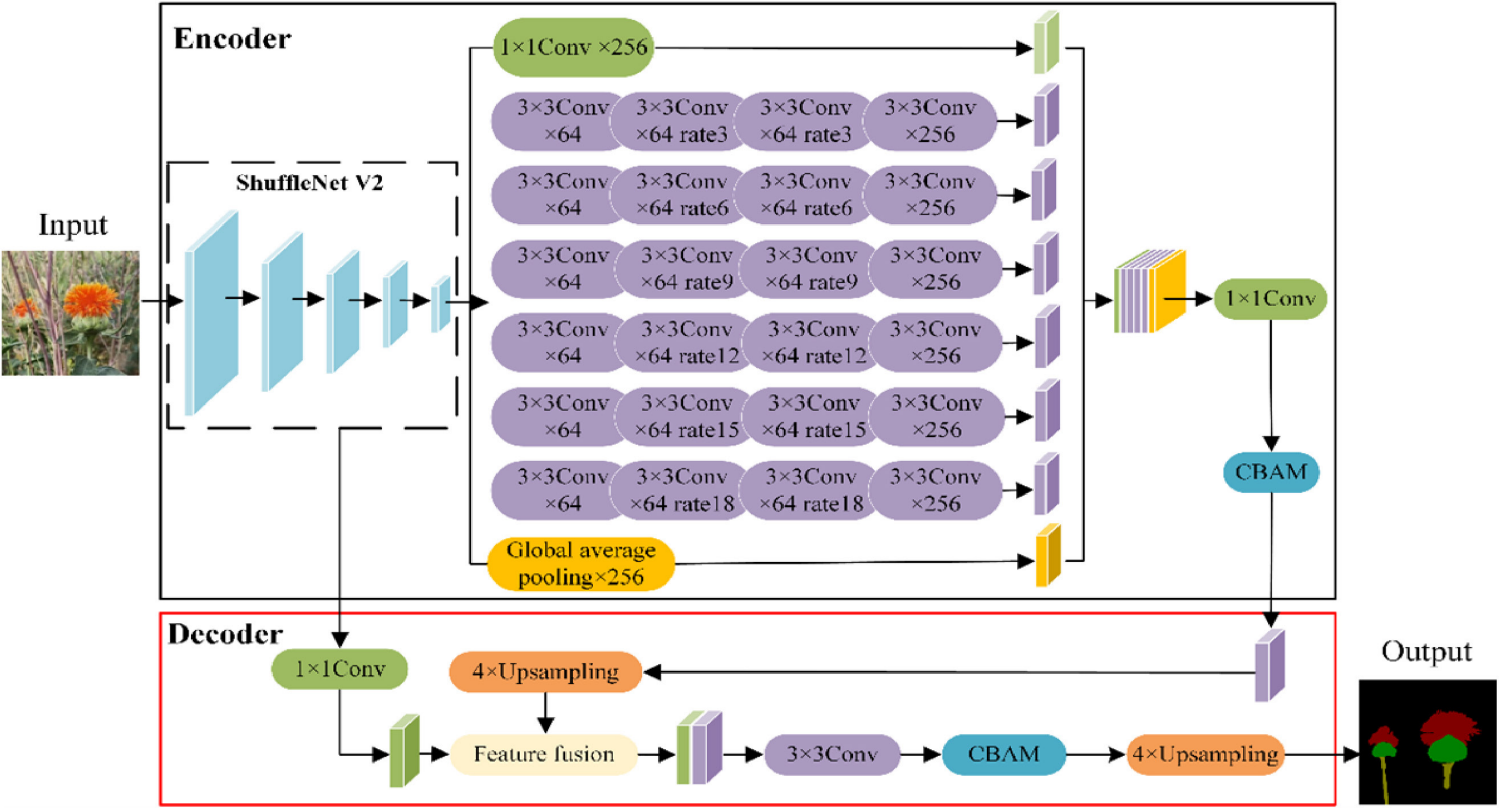
Schematic of the SDC-DeepLabv3+ algorithm structure.

To reduce the number of algorithm parameters, further capture the global and contextual information, and avoid the localization accuracy deviation caused by missed segmentation and mis-segmentation, a method for safflower filament picking-point localization with improved DeepLabv3+ is proposed (SDC-DeepLabv3+), which fuses the modules of ShuffletNetV2 [33], the ASPP of dilated depth-separable convolution (DDSC-ASPP), and CBAM [42], as shown in Fig. 3. To distinguish DeepLabv3+, the SDC-DeepLabv3+ algorithm is reordered with the initials of ShuffletNetV2, DSC-ASPP, and CBAM as content combinations. Lightweight ShuffleNetV2 is used as the backbone network. The ASPP is improved to DDSC-ASPP, extracting more safflower features from different receptive fields. Moreover, CBAM is added so that the fusion of deep and shallow safflower features is allocated to the channel and spatial-attention resources. The feature information is maximized. SDC-DeepLabv3+ not only reduces the computing cost, but also considers the segmentation accuracy. This improves the capability of the algorithm to predict the localization accuracy of the safflower-filament picking points.

#### ShuffletNetV2 module

**Fig. 4.**
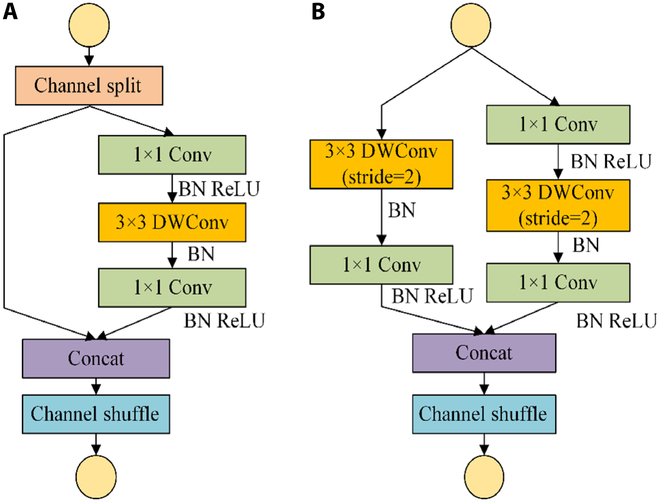
Structure of ShuffleNetV2. (A) Stride = 1 block and (B) stride = 2 blocks.

To reduce algorithm computation and improve detection accuracy, the ShuffleNetV2 [32] structure is selected as the backbone network, as shown in Fig. 4. ShuffleNetV2 is based on ShuffleNetV1 [43] and retains the components of ShuffleNetV1, such as the channel shuffle and depth-separable convolution. A more efficient basic block is proposed to reduce the number of algorithm parameters and computations and improve detection accuracy.

The input safflower image was first subjected to 24 standard convolutions of size 3 × 3 and step 2 for feature extraction to obtain a new feature map. MaxPool was then used to maximize pooling for downsampling purposes to obtain a 24 × 56 × 56 feature map. Finally, 3 stage structures consisting of ShuffleNetV2 basic components with stride = 1 and 2 blocks were connected [44]. The semantic information of the different layers of the target image was extracted.

Each unit feature channel input of the component stride = 1 block was divided into 2 branches, one of which was changed, and the other comprised 3 convolutions. The 2 1 × 1 convolutions were no longer group convolutions but ordinary 1 × 1 convolution operations. After convolution, the 2 branches were connected and fused. The number of channels was maintained. A channel shuffle operation was performed to enable information communication between the 2 branches, where the connected fusion of the channel shuffle and channel split of the next modular unit were synthesized into an element-level operation.

For spatial downsampling, the stride = 2 block removed the channel-split operator and channel split, and each branch copied the input directly. Each branch had stride = 2 downsampling for concatenated fusion [31]. The filament feature-map space size was halved, but the number of channels doubled. Setting the number of channels in each block, such as 0.5× and 1×, can adjust the complexity of the model. The specific structure is shown in Fig. 4. The architecture was designed with high efficiency to realize information communication between different channel groups and improve the accuracy of filament feature extraction.

#### DDSC-ASPP

Compared with other detection objects [24–26], the shapes and distributions of safflower filaments, fruit balls, and branches are unstable, varied, and susceptible to occlusion. The DeepLabv3+ algorithm uses an ASPP module with a combination of dilation rates of 6, 12, and 18 to extract multiscale features [45,46]. However, its large dilation rate leads to a large receptive field, insufficiently detailed feature extraction, and poor extraction of small targets. It is difficult to capture all types of information in a single-scale receptive field [47]. Therefore, combined with the edge complexity of the safflower features and the high leakage rate of small targets, this study added 3 dilated depth-separable convolution [48] branches based on the ASPP module. The filament, fruit ball, and branch feature information are extracted under the receptive field using depth-separable convolution with higher sampling rates. 

1. Depth-separable convolution

**Fig. 5.**
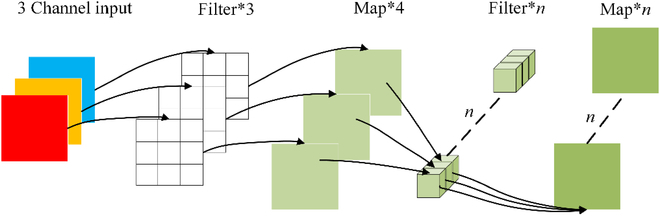
Comparison of depth-separable convolution processes.

By retaining the original feature extracted from the receptive field, the depth-separable convolution actively reduces the weights and algorithm training [30]. The convolution process is divided into 2 parts: channel-by-channel and point-by-point convolution. The convolution kernel of the channel-by-channel convolution corresponds one-to-one to the channel of the input image. After the channel-by-channel convolution, the number of channels for the feature map remained the same as that before the input. Because the same locations of different channels had connections, a 1 × 1 point-by-point convolution was used to adjust the number of channels and establish connections between them. A schematic of the depth-separable convolution is shown in Fig. 5.

2. Increasing separable convolution of the dilated depth at different sampling rates

The ASPP structure comprises a dilated convolution layer with different sampling rates, a 1 × 1 convolution layer, and an image-pooling layer [6]. However, because of the large sampling-rate interval of the dilated convolution, the feature extraction of the ASPP structure was more sparse, resulting in the loss of some local information from the safflower image [49,50]. Therefore, 3 dilate depth-separable convolution branches were added, based on the original sampling rate. The combination of the dilate rate was modified to 3, 6, 9, 12, 15, and 18. The middle 6 convolutional branches were subjected to 2 3 × 3 convolutional cascade operations. A dilated depth-separable convolution that realizes multiscale feature extraction can be added to the receptive field.

When the dilation rate of the dilated depth-separable convolution is *r*, and the size of the convolution kernel is *k*, the size of the receptive field is as follows:

$$R=\left(r-1\right)\left(k-1\right)+k$$ (1)

However, when the 2-layer dilated depth-separable concentration series is cascaded, the receptive field size is as follows:

$$R=R_{1}+R_{2}-1$$ (2)

where *R*_1_ and *R*_2_ denote the receptive fields provided by the 2-layer dilated depth-separable convolution.

DDSC-ASPP first reduced the middle 4 convolutional branches to 64 channels using a 1 × 1 convolution. Two 3 × 3 convolutions were then performed. Subsequently, using 1 × 1 convolution dimensions for 256 channels, the bottleneck structure effectively reduced the parameters. The DDSC-ASPP decreased the loss rate of edge information and small-target safflower information in the safflower image. This can facilitate detailed feature extraction of small targets and safflower contour edges. Thus, the problem of missing the segmentation of the filament, fruit ball, and branch was effectively minimized.

#### CBAM

To accurately extract edge features and solve the interference problem of background information for filaments, fruit balls, and branches, this study proposes injecting the CBAM into the feature-extraction module of the encoding and decoding layers [51,52].

**Fig. 6.**
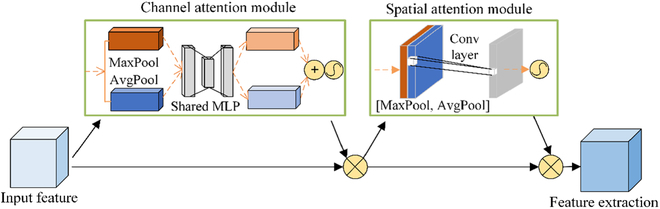
CBAM structure.

Unlike Squeeze-and-Excitation [53] and Efficient Channel Attention [54], which consider channels and ignore spatial information, CBAM is a lightweight module that combines channel and spatial attention mechanisms [55]. Its core aim is to provide segmentation attention to the desired region, as shown in Fig. 6. Given a safflower-feature map, the CBAM was able to sequentially generate attention feature-map information in both channel and spatial dimensions. The channel-attention mechanism was operated first, followed by the spatial-attention mechanism. Then, the information of the 2 feature maps was multiplied by the original input feature map for adaptive feature correction to produce the final feature map. The CBAM was embedded into the backbone network to improve performance and achieve an enhanced ROI in both the channel and spatial dimensions.

The CBAM captured the correlation between features in different dimensions by adapting learning channels and spatial-attention weights. The enhanced features were used as inputs for the subsequent network layers. It suppressed safflower-image noise and irrelevant information while preserving key information. Therefore, the performance of safflower recognition and detection was enhanced, realizing the accurate segmentation of filaments, fruit balls, and branches.

### Localization method for filament picking point

**Fig. 7.**
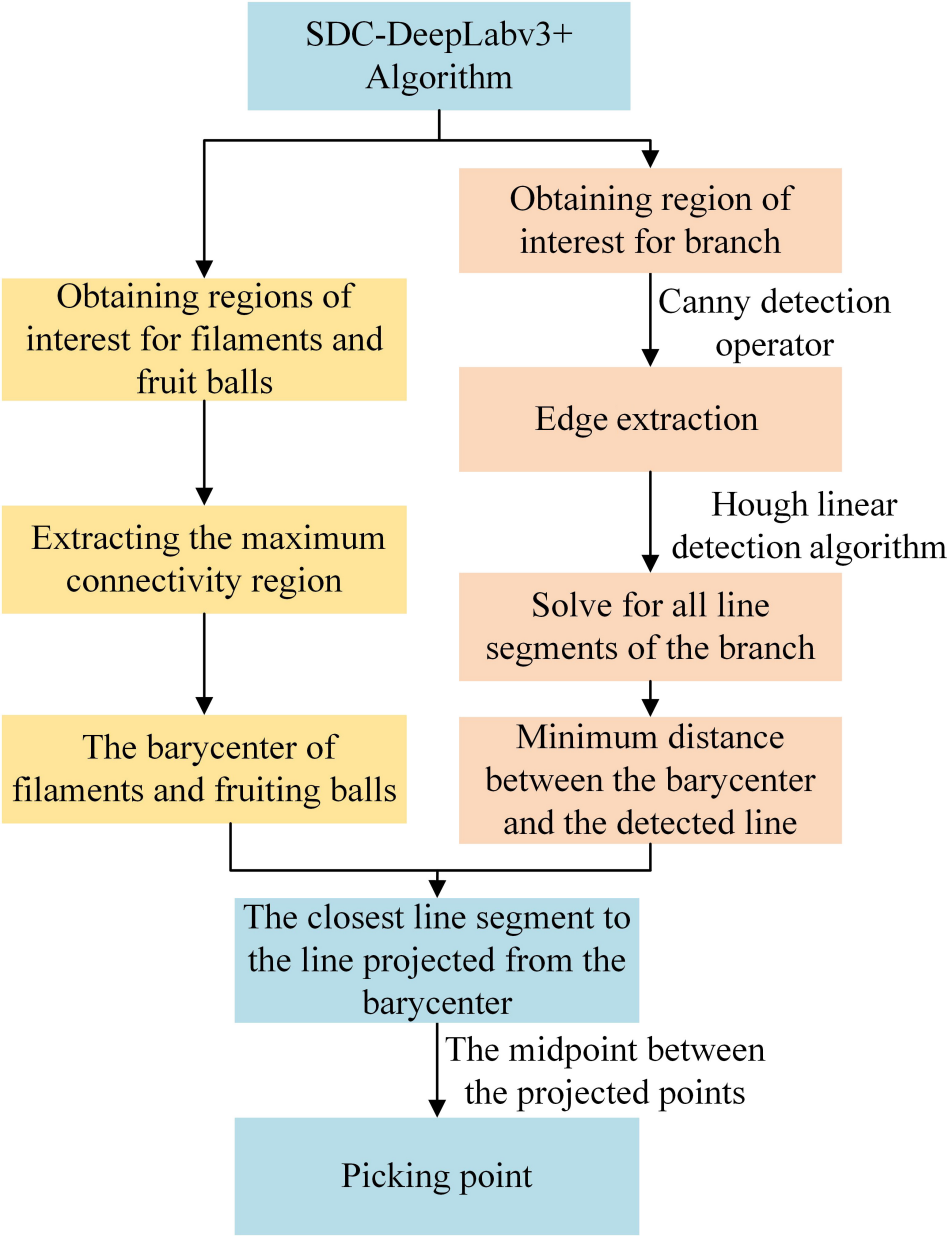
The flowchart of the filament picking-point localization method.

To improve the localization accuracy and speed of damage-free safflower-filament picking and remove the influence of image noise on the localization of the picking point, combined with the characteristics of safflower-growth diversity, this study set up an ROI in the branch region [56]. The flowchart of the filament picking point localization method is shown in Fig. 7.

**Fig. 8.**
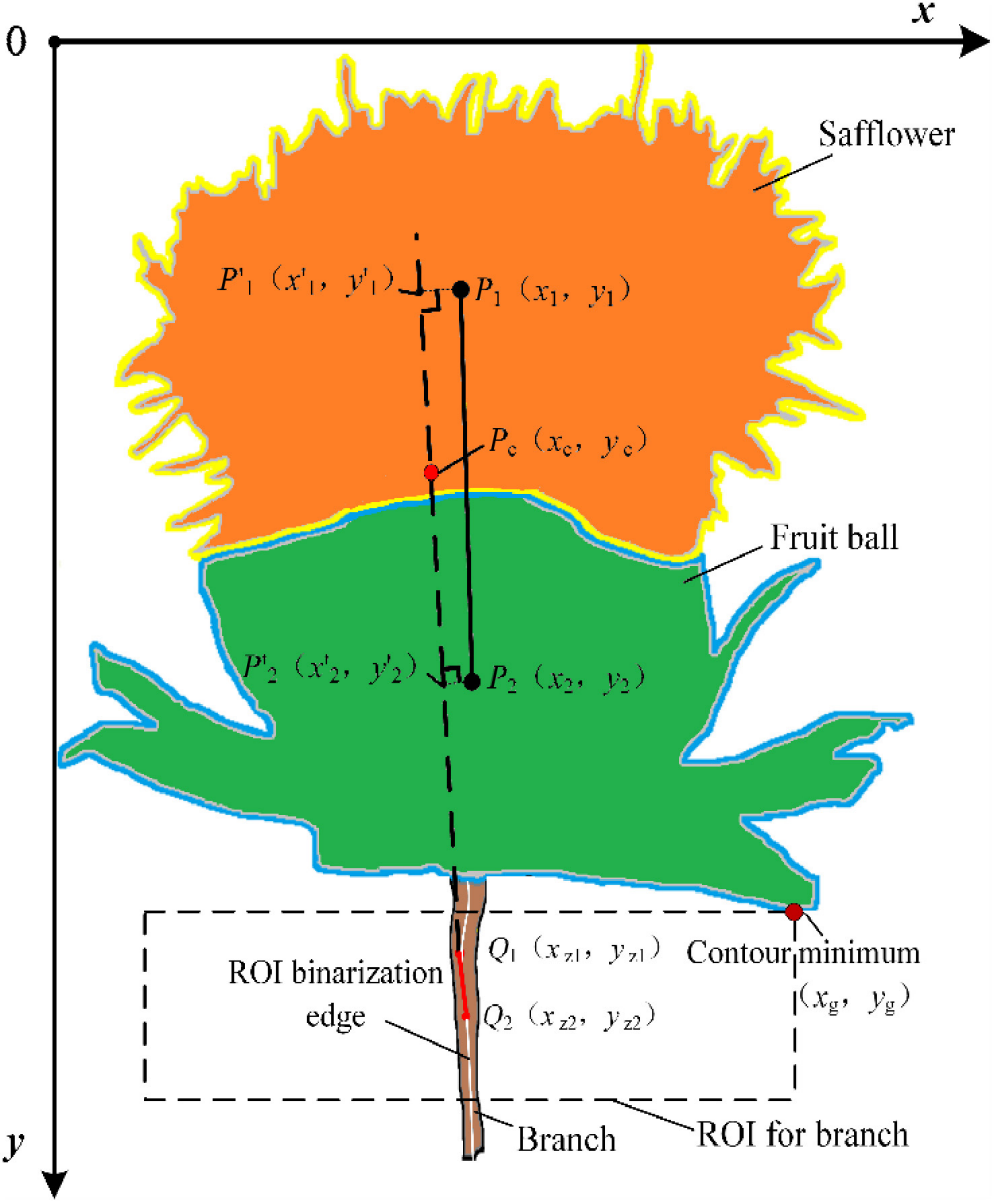
Schematic of picking-point calculation model. The barycenter coordinates of the filament and fruit ball are *P*_1_ (*x*_1_, *y*_1_) and *P*_2_ (*x*_2_, *y*_2_), respectively. The barycenter projection of the filament and fruit ball are *P*′_1_ (*x*′_1_, *y*′_1_) and *P*′_2_ (*x*′_2_, *y*′_2_), respectively. The values of the 2 endpoint coordinates are *Q*_1_ (*x*_z1_, *y*_z1_) and *Q*_2_ (*x*_z2_, *y*_z2_), respectively. The value of the picking point coordinates is *P*_c_ (*x*_c_, *y*_c_).

Firstly, the safflower image was preprocessed according to the SDC-DeepLabv3+ algorithm and segmented into filaments, fruit bulbs, branches, and trunks. The interest regions of filaments and fruit bulbs were obtained. The maximum connectivity region of the two was extracted, and the center of mass coordinates of the filament and fruit ball were solved. Meanwhile, edge detection was performed on the ROI based on the branch ROI obtained by the SDC-DeepLabv3+ algorithm to obtain an edge binary map. All the line segments in the binary map were solved using the Hough line-detection algorithm [57]. The line segment that solved for the minimum distance between the barycenter coordinates of the filament and fruit ball and the line was considered as the line position of the picking point and was projected onto this line segment. Finally, the midpoint of the 2 barycenters for the projection points on the line section was used as the picking point. A schematic of the picking-point calculation model is shown in Fig. 8.

#### ROI extraction with linear detection

Safflower grows naturally upward. In the absence of interference from other supports, safflower filaments open on the fruit ball, and the branch is typically located directly below the filament and fruit ball. To minimize environmental interference with the localization of the picking point, the ROI of the branch was determined based on the barycenter of the filament, barycenter of the fruit ball, lowest point of the contour, and distance between the left and right poles, as shown in Fig. 8.

In the ROI images, the edge response of the branches was stronger. To simultaneously suppress noise and enhance the boundary, a safflower image was subjected to a binarization operation [58]. The barycenter coordinates of the filament *P*_1_ (*x*_1_, *y*_1_) and fruit ball *P*_2_ (*x*_2_, *y*_2_) were derived. The edge gradient-detection algorithm was used for edge extraction of the branch ROI. The gradient-direction vector was calculated using a finite difference. The gradient of the pixel points in the ROI image was calculated as follows:

$$\begin{array}{c} \left\{\begin{array}{c} \begin{array}{c} ||\nabla f(x,\;y)||={\left\{f_{w}{\left(x,\;y\right)}^{2}+\;f_{h}{\left(x,\;y\right)}^{2}\right\}}^{1/2}\\ f_{w}(x,\;y)=\frac{1}{2}\left(f(x+1,\;y)-f(x,-1\;y)\right)\\ f_{h}(x,\;y)=\frac{1}{2}\left(f(x,\;y+1)-f(x,\;y-1)\right) \end{array} \end{array}\right. \end{array}$$ (3)

where *f*(*x*, *y*) denotes the pixel-point value in the ROI image, and *f*_w_(*x*, *y*) and *f*_h_(*x*, *y*) denote the gradients in the direction of the horizontal and vertical coordinate axes, respectively.

The local maximum points of the gradient are considered as the corresponding edge pixel points in the safflower image. Subsequently, the generated gradient map was thresholded for image segmentation to obtain a binary edge map of the ROI of the branch.

#### Picking-point localization based on barycenter projection

Because the edges of the branches were presented as lines, the Hough line-detection algorithm was used to detect the lines in the binary edge map within the branch ROI. All the line segments in the region that satisfied these conditions were detected. The values of the 2 endpoint coordinates *Q*_1_ (*x*_z1_, *y*_z1_) and *Q*_2_ (*x*_z2_, *y*_z2_) were recorded for all detected line sections, as shown in Fig. 8. Based on the coordinates of the 2 endpoints, the equation for the line is as follows:

$$\frac{x-x_{z1}}{x_{z2}-x_{z1}}=\frac{y-y_{z1}}{y_{z1}-y_{z2}}$$ (4)

where (*x*, *y*) are the pixel coordinates, and (*x*_z1_, *y*_z1_) and (*x*_z2_, *y*_z2_) are the coordinates of the first and second endpoints of the branch line, respectively.

The distance from the barycenter of the filament and fruit ball to each line *L*_b_ was calculated based on the coordinates of the endpoints for all satisfied lines. The minimum constraint function for the distance between the barycenter and line min (*L*_b_) was solved to determine the line with the minimum distance from the picking point. The line distance *L*_b_ to the center of mass is calculated as follows:

$$L_{b}=\frac{\left|\frac{y_{z1}-y_{z2}}{x_{z1}-x_{z2}}x_{b}+\frac{x_{z1}y_{z2}-y_{z1}x_{z2}}{x_{z1}-x_{z2}}-y_{b}\right|}{\sqrt{1+{\left(\frac{y_{z1}-y_{z2}}{x_{z1}-x_{z2}}\right)}^{2}}}$$ (5)

Because safflower naturally grows upward and opens flowers on the fruit ball, the branch is typically located directly below the barycenter. Theoretically, the extension line through the branch must pass through the barycenter. However, under the influence of image-segmentation errors, blurred and irregular contours of the filament and fruit ball, and other disturbing factors in practice, the calculated extension of the branch line tends to be offset from the barycenter to a certain extent. The barycenter projection of the filament and fruit ball, *P*′_1_ (*x*′_1_, *y*′_1_) and *P*′_2_ (*x*′_2_, *y*′_2_), is obtained by calculating the barycenter-projection point on the line, where the picking point is located at the minimum distance using Eq. 5. The midpoint between the 2 barycenter projection points on the line was considered as the picking point *P*_c_ (*x*_c_, *y*_c_).

## Results

### Test platform

The training platform used in this study was a Windows 11 (64-bit) operating system (Lenovo R9000X, Razor 8-core R7-5800H, RTX3060). The computer was equipped with an Intel(R) Xeon (R)CPUE52630v4@2.20 GHz operating memory, 64 GB running memory, 1 TB SSD, and 12 GB GTX1080Ti×2 GPU. Anaconda 3.5.0 (Anaconda Inc., USA), CUDA 11.0 (Nvidia, USA), and cuDNN 6.0 (Nvidia, USA) libraries were used. In addition, the open-source deep learning framework Pytorch was used as the development environment, and the programming language used was Python 3.9 (Python Software Foundation, USA).

### Evaluation indicators

In this study, mean pixel accuracy (mPA), mean intersection over union (mIoU), frames per second (FPS), and params were used as algorithm performance-evaluation metrics. mPA calculates the number of pixels that are correctly classified in each pixel category within a class and then determines the average of all classes, %. The mIoU is a standard metric for semantic segmentation. It calculates the ratio of the intersection and concatenation of the set of true and predicted values [59]. The evaluation metrics are calculated as follows:

$$\text{mPA}=\frac{1}{k+1}\sum_{i=0}^{k}\frac{p_{\mathrm{ii}}}{\sum_{j=0}^{k}p_{\mathrm{ij}}}$$ (6)

$$\text{mIOU}=\frac{1}{k+1}\sum_{i=0}^{k}\frac{p_{\mathrm{ii}}}{\sum_{i=0}^{k}p_{\mathrm{ij}}+\sum_{i=0}^{k}p_{\mathrm{ji}}-p_{\mathrm{ii}}}$$ (7)

$$\mathrm{MP}=\frac{1}{k+1}\sum_{i=0}^{k}\frac{p_{\mathrm{ii}}}{\sum_{j=0}^{k}p_{\mathrm{ii}}+\sum_{j=0}^{k}p_{\mathrm{ij}}}$$ (8)

$$\mathrm{MR}=\frac{1}{k+1}\sum_{i=0}^{k}\frac{p_{\mathrm{ii}}}{\sum_{j=0}^{k}p_{\mathrm{ji}}-p_{\mathrm{ii}}}$$ (9)

where *p_ii_* denotes the number of true instances, while *p_ij_* and *p_ji_* are interpreted as false-negative and false-positive instances, respectively. False-negative instances are samples predicted to be non-filament, fruit ball, and branch regions and actual filament, fruit ball, and branch regions. False-positive instances are samples predicted to be filament, fruit ball, and branch regions and actual non-filament, fruit ball, and branch regions; *k* denotes category.

### Localization performance test

A test set of images was used to validate the performance of the SDC-DeepLabv3+-based picking-point localization method. A target safflower was selected from each test image by manual labeling, and the coordinate position of the best picking point was recorded. In the test, SDC-DeepLabv3+ was used to process the safflower images of the test, and the 2-dimensional coordinate values of the predicted target picking points were recorded. These errors were used as an important basis for judging the performance of the picking-point localization method [56]. The prediction accuracy of the picking-point localization method was evaluated using pixel error. The formula for the pixel error is as follows:

$$\left\{\begin{array}{c} e=\sqrt{\left({e_{x}}^{2}+{e_{y}}^{2}\right)}\\ e_{x}=\text{min}\left|X_{\mathrm{bt}}-x\right|\\ e_{y}=\text{min}\left|Y_{\mathrm{bt}}-y\right| \end{array}\right.$$ (10)

where *X*_bt_ and *Y*_bt_ denote the horizontal and vertical coordinate values of the manually labeled best picking point in the image, respectively; *x* and *y* denote the horizontal and vertical coordinate values of the picking point predicted by the picking-point localization method in the image, respectively; *e*_x_ and *e*_y_ denote the pixel-point error value between the predicted and manually labeled picking points in the *x*/*y* direction; and *e* denotes the combined pixel-point error value of the predicted and manually labeled picking points.

The 2-dimensional coordinate values of the filament picking point were mapped and matched with the 3-dimensional depth map acquired by a camera (RealSense D435i, Intel, USA). Using the inherent internal parameters of the depth camera, the image coordinate system was converted to the camera’s optical coordinate system to obtain the 3-dimensional spatial coordinate values of any coordinate point in the camera coordinate system. Subsequently, the global coordinate system was set on a calibration plate. The first square was used as the reference point in the upper-left corner of the calibration plate and set as the origin of the global coordinate system. The normal direction of the calibration-plate plane was used as the *Z*-axis depth direction, and the horizontal direction was used as the *X*-axis direction. The 3-dimensional coordinates of the filament picking point were obtained by interconverting the camera coordinate system with the global coordinate system of the harvesting robots in the global coordinate system. By comparing the results from the manual measurement and depth camera, the visual localization error *E*_Z_ in the *Z* direction and localization error *E*_X_ in the *X* direction were calculated as follows:

$$E_{Z}=\frac{\sum_{m=1}^{M}\left|Z{\left(\mathrm{CP}\right)}_{m}-Z{\left(\mathrm{GT}\right)}_{m}\right|}{M}$$ (11)

$$\begin{array}{c} E_{X}=\frac{\sum_{m=1}^{M}\left|X{\left(\mathrm{CP}\right)}_{m}-X{\left(\mathrm{OP}\right)}_{m}-L_{m}\right|}{M} \end{array}$$ (12)

where *M* and *m* denote the total number of image combinations and image sequence number, which are *M* = 50 and *m* [1, 50], respectively; *Z*(*CP*)_m_ and *X*(*CP*)_m_ denote the values of the *Z* and *X* coordinates of the picking point measured by the depth camera, respectively; *Z*(*GT*)_m_ denotes the value of the *Z* coordinates of the manually measured picking point; *X*(*OP*)_m_ denotes the value of the *X* coordinates of the calibration-plate reference point (the origin of the global coordinate system); and *L*_m_ denotes the manually measured distance between the calibration-plate reference point and filament picking point.

## Algorithm training results

**Fig. 9.**
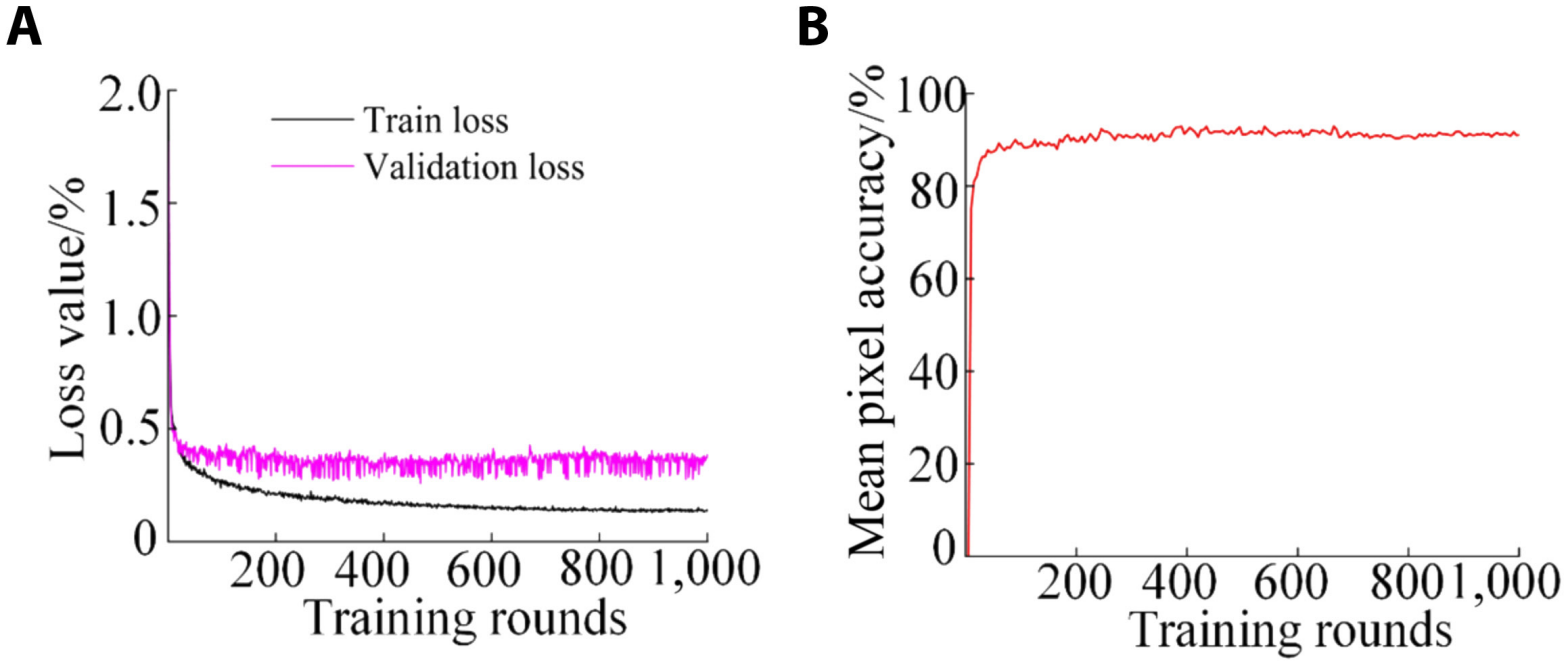
Parameters of the training process. (A) Loss curve and (B) mean pixel accuracy.

To improve algorithm performance and decrease overfitting, the initial learning rate of the SDC-DeepLabv3+ algorithm was 0.01, the batch size was set to 8, and the number of training iterations was 1,000. If the accuracy was not increased within 15 rounds of training, the learning rate would be reduced to 0.01 times the original one using the SGD optimizer [42]. The network-weight file was saved after every 10 training epochs. The weights with the highest and lowest accuracies were saved. The algorithm was validated using a test set, and the detection results were output. The training results for the SDC-DeepLabv3+ algorithm are shown in Fig. 9.

The loss value decreased the fastest in the first 163 rounds of the training process. It was still decreasing after 163 rounds, and the rate gradually became slower. When the number of iterative training rounds reached 902, the loss curve tended to flatten out. It eventually stabilized at the minimum value and completed the training, as shown in Fig. 9A. Meanwhile, the mPA curve gradually rose with the increase of training rounds and finally stabilized. As shown in Fig. 9B, the mPA (IoU = 0.5) is 92.61%, which indicates that the algorithm converges. The trend of each curve during the training process can reflect the effectiveness of training. It is favorable to improve the accuracy of safflower segmentation.

## Evaluation of the filament-segmentation algorithm

### Ablation test

Four sets of ablation tests were conducted to verify the effects of the ShuffletNetV2 backbone network, DDSC-ASPP, and CBAM on the recognition accuracy of the SDC-DeepLabv3+ semantic segmentation algorithm. The test results are listed in Table 1.

**Table 1.** Results of the ablation test

Algorithms	Backbone networks	Pyramid module	Add module	mIoU/%	mPA/%	FPS/(f·s^−1^)	Weights file size/MB
DeepLabv3+	Xception	ASPP	None	94.13	95.64	45.32	206.32
DeepLabv3+	ShuffletNetV2	ASPP	None	95.01	96.33	56.19	22.75
DeepLabv3+	ShuffletNetV2	DDSC-ASPP	None	95.35	96.52	58.24	24.61
DeepLabv3+	ShuffletNetV2	DDSC-ASPP	CBAM	95.84	96.87	57.48	25.54

The backbone network of the original DeepLabv3+ was Xception. The mIoU of this algorithm for safflower detection was 94.13% and the mPA was 95.67%. After changing the backbone network of DeepLabv3+ from Xception to ShuffletNetV2, the mIoU and mPA improved by 0.88 and 0.69 percentage points, respectively. The weights file size was reduced significantly. When ASPP was improved to DDSC-ASPP, mIoU and mPA were enhanced by 0.34 and 0.19 percentage points, respectively. This shows that DDSC-ASPP enriched the expression of effective features and enhanced the detection of filaments for small targets. To a certain extent, the algorithm-segmentation accuracy was improved, and the detection effect was enhanced. Finally, with the introduction of the CBAM, the SDC-DeepLabv3+ algorithm enhanced the mIoU and mPA to 95.84% and 96.87% for safflower detection, respectively. This significantly improved the linkage of filament features in the channel and space, suppressed background interference, and highlighted more safflower filaments. In addition, the FPS decreased marginally with the addition of DDSC-ASPP alone. 

Comprehensive ablation tests showed that SDC-DeepLabv3+ significantly improved the mIoU, mPA, and FPS. The weights of the improved network algorithms were significantly reduced. Therefore, the safflower features refined by the SDC-DeepLabv3+ algorithm received attention weights in the channel and spatial dimensions and more prominent target edge features. This provides support for the fast and accurate localization of the picking points.

### Comparison of different algorithms with backbone networks

To verify the effectiveness of the feature-extraction network and further analyze the segmentation performance of the improved DeepLabv3+, 5 segmentation algorithms, including PSPNet [24], SegNet [25], U-Net [26], DeepLabv3+ [33], and SDC-DeepLabv3+, were comparatively tested under the same guarantee of other parameters, as shown in Table 2.

**Table 2.** Comparison of different segmentation algorithms and backbone networks

Algorithms	Backbone networks	mIoU/%	mPA/%	FPS/(f·s^−1^)	Weights file size/MB
PSPNet	ResNet50	92.86	94.99	48.37	176.53
U-Net	ResNet50	93.15	95.13	39.65	92.07
SegNet	ResNet50	92.69	94.66	47.11	24.26
DeepLabv3+	Xception	94.13	95.64	45.32	206.32
SDC-DeepLabv3+	ShuffletNetV2	95.84	96.87	57.48	25.54

1. Performance comparison of different segmentation algorithms

**Fig. 10.**
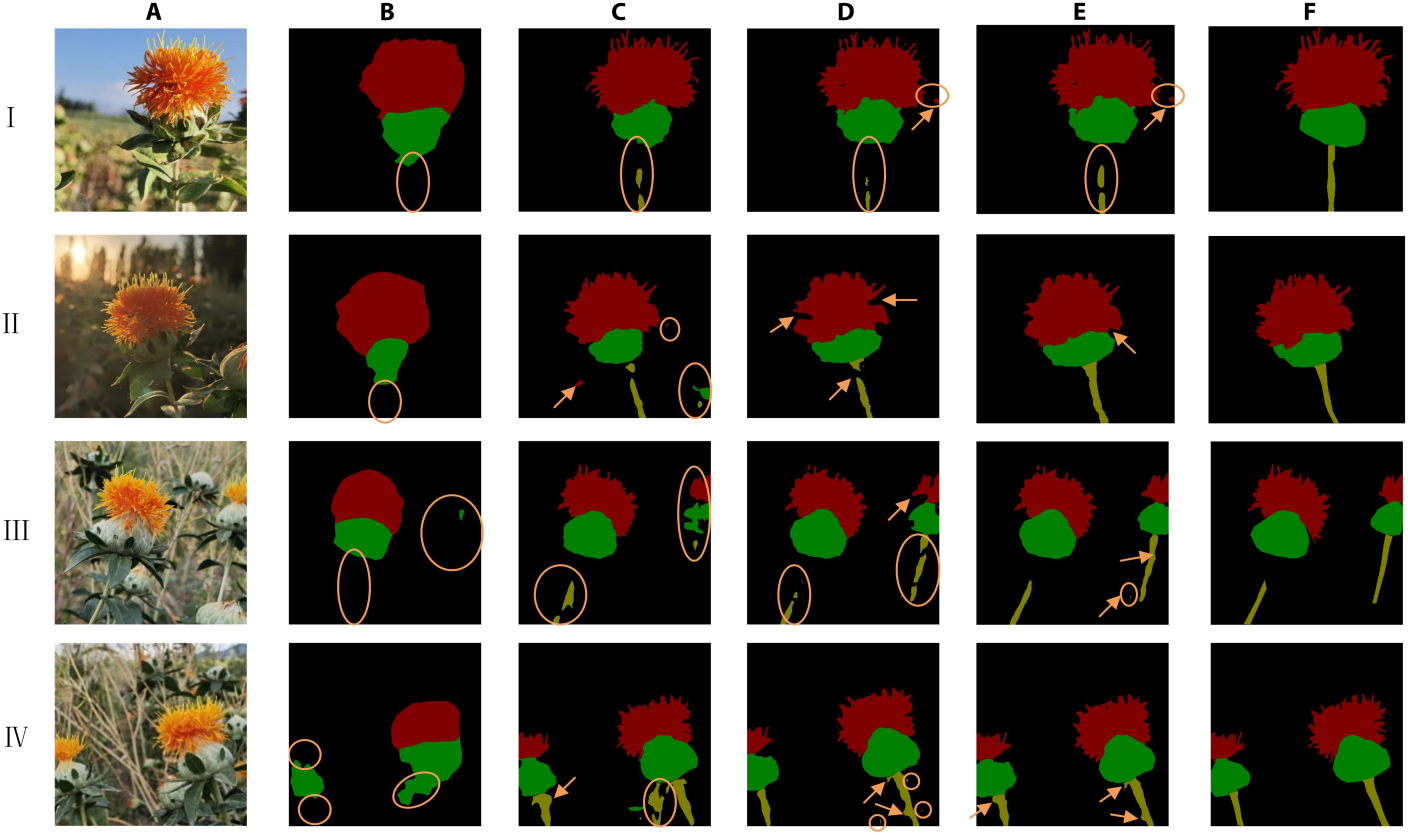
Detection results of different segmentation algorithms. (I) Sunny day with light, (II) sunny day with backlight, (III) overcast day with light, and (IV) cloudy day with light. (A) Original image, (B) resulting image of PSPNet, (C) resulting image of U-Net, (D) resulting image of SegNet, (E) resulting image of DeepLabv3+, and (F) resulting image of SDC-DeepLabv3+. The arrows indicate areas in which depressions or burrs were detected, and the circles indicate areas in which segmentation errors or omissions were detected.

As shown in Table 2, the PSPNet algorithm failed to completely detect the safflower filaments and branches (Fig. 10B). The mIoU and mPA values for the U-Net algorithm were 93.15% and 95.12%, respectively. The detection results were unclear, and the algorithm could not effectively address the irregular and blurred contours of the branches (Fig. 10C). By comparison, the mIoU and mPA values of the SegNet algorithm were 0.46% and 0.47% lower, respectively. The detection results were consistent; however, sporadic regions were incorrectly detected (Fig. 10D). The DeepLabv3+ algorithm used cavity convolution and a multi-scale strategy to significantly increase the receptive field; however, depressions and burrs remained (Fig. 10E). The accuracy of safflower segmentation was better than that of the previous 3 algorithms, but the prediction speed may be improved. After replacing the backbone network of DeepLabv3+ with ShuffletNetV2, SDC-DeepLabv3+ effectively decreased the number of weights and improved the prediction speed. Relative to DeepLabv3+, the mIoU and mPA improved by 1.71% and 1.23%, respectively, and the FPS improved by 12.16 f/s. This was because the filament was characterized, and the fruit balls and branches differed from the background region. However, SDC-DeepLabv3+ was more capable of detecting small targets with the accurate extraction of edge features. The interference problem of the background information for the filament, fruit ball, and branch feature extraction was further solved, as shown in Fig. 10F. This proved that the improved algorithm achieved excellent results in terms of both segmentation accuracy and prediction speed, making it suitable for deployment in embedded robots. The algorithm was guaranteed to have high segmentation accuracy while being lightweight.

2. Performance comparison of different backbone networks

As shown in Table 2, the accuracy of SDC-DeepLabv3+ for safflower segmentation was superior to that of the previous 4 groups of algorithms. The mIoU and mPA improved, the number of model weights decreased, and the prediction speed improved. In terms of image-segmentation performance, the mIoU of ShuffletNetV2 was able to achieve 95.84%, which was 2.69 and 1.71 percentage points higher than those of ResNet50 and Xception, respectively. The mPA was 96.87%, which was 1.75 and 1.23 percentage points higher than those of ResNet50 and Xception, respectively. In addition, different backbone networks could efficiently segment the safflower images. ShuffletNetV2 exhibited an improvement of at least 9.11 and 12.16 f/s over ResNet50 and Xception, respectively. The segmentation effectiveness and training efficiency showed that the lightweight ShuffletNetV2 feature-extraction network was effective and could be used as a backbone network for the safflower image-segmentation algorithm.

3. Performance comparison of 4 different weather and lighting environments

Comparative results of the SDC-DeepLabv3+ algorithm and DeepLabv3+ algorithm for MP, MR, mIoU, and mPA under 4 different weather and lighting environments are shown in Table 3.

**Table 3.** Results of algorithm under 4 different weather and lighting environments

Algorithms	Evaluation indicators	Weather and lighting			
		Sunny day with light	Sunny day with backlight	Overcast day with light	Cloudy day with light
DeepLabv3+	*MP*/%	93.12	92.58	90.63	91.12
	*MR*/%	85.47	84.84	82.91	84.05
	mIoU/%	93.61	91.70	90.28	91.13
	mPA/%	93.78	92.35	90.05	90.65
SDC-DeepLabv3+	*MP*/%	95.16	94.08	90.95	93.84
	*MR*/%	87.27	86.60	85.19	86.02
	mIoU/%	95.07	94.57	91.26	93.57
	mPA/%	95.50	94.61	91.35	92.72

As shown in Table 3, the segmentation accuracy of the SDC-DeepLabv3+ algorithm was better than the DeepLabv3+ algorithm for different weather and lighting environments. The SDC-DeepLabv3+ algorithm showed the best performance in the evaluation indicators MP, MR, mIoU, and mPA under sunny day with light, which all outperformed the sunny day with backlight and overcast day with light by at least 0.32%, and improved the performance relative to the DeepLabv3+ algorithm by 2.04%, 1.80%, 1.46%, and 1.72%, respectively. It indicated that the present algorithm was better in sunny conditions than in cloudy and cloudy conditions, which were more affected by light. Especially in the low-light environment, the algorithm was difficult to distinguish safflower color and texture features, and mis-segmentation occurs, as shown in Fig. 10.

## Localization performance test

**Fig. 11.**
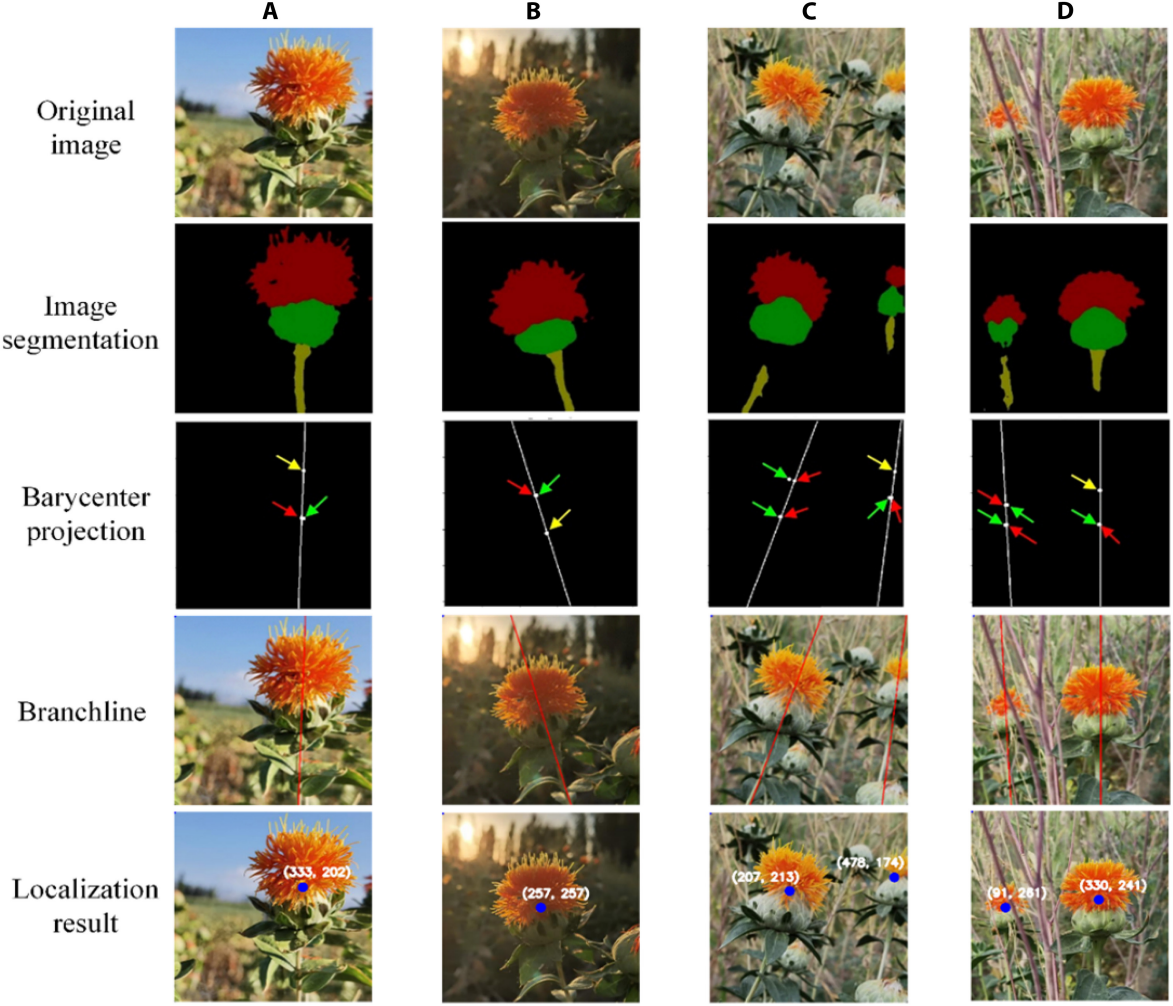
Location process of picking points with 4 types of lighting. (A) Sunny day with light, (B) sunny day with backlight, (C) overcast day with light, and (D) cloudy day with light. Green arrows indicate the barycenter points of the safflower filaments or fruit balls; red arrows are projection points of the safflower filament or fruit ball barycenter; and yellow arrows are barycenter points coinciding with projection points.

The localization accuracy of SDC-DeepLabv3+ at the filament picking point was verified under 4 different weather conditions. The results of the filament picking-point localization are shown in Fig. 11. Four images were selected to test the pixel coordinates and localization errors of the picking points, as shown in Fig. 11 and Table 4.

**Table 4.** Picking point and pixel-localization errors

Types	The barycenter coordinates of filament/pixel	The barycenter coordinates of the fruit ball/pixel	Picking point/pixel	Pixel localization error/pixel		
	(*X*_1_, *Y*_1_)	(*X*_2_, *Y*_2_)	(*x*, *y*)	*e*_x_	*e*_y_	*e*
Sunny day with light	(336, 135)	(335, 265)	(333, 199)	0	0	0
Sunny day with backlight	(244, 206)	(270, 311)	(256, 258)	0	2	2
Overcast day with light	(489, 143)	(475, 214)	(484, 178)	0	1	1
	(213, 162)	(189, 265)	(209, 216)	0	5	5
Cloudy day with light	(331, 194)	(330, 287)	(329, 240)	0	2	2
	(89, 234)	(84, 289)	(86, 261)	1	0	1

The test results did not exhibit an error between the picking point and optimal picking point obtained by the proposed method on a sunny day with a backlight. The localization accuracy was marginally higher than that of the backlight. The optimal picking-point pixel range was manually set. The branch center axis was used as the location of the optimal picking point (generally a rectangular range of 30 × 10 pixels). Owing to the changes in lighting conditions, sunny days with light improved the light intensity and brightness on the safflower, improving the localization of the picking point. However, the error between the obtained and optimal picking points was 2 pixels greater in backlight than on a sunny day with light. The backlighting effect increased the intensity and brightness of the safflower, but the filament blended with the surrounding complex environment.

However, the glare from the backlight caused the filament to overlap with the background color, resulting in the localization of safflowers with the same color as the surrounding background. The pixel error in the row direction was small, and the optimal picking point was between cloudy and sunny conditions with light. Moreover, 1 to 5 pixel errors occurred between the column direction and optimal picking point. In particular, the pixel error was largest for cloudy light in the direction of the column. The main reason for this was that the light intensity on cloudy days was weak compared with that on overcast days; thus, the color of the picking-point site was not prominent. Therefore, the localization of the picking point was prone to deviation.

**Fig. 12.**
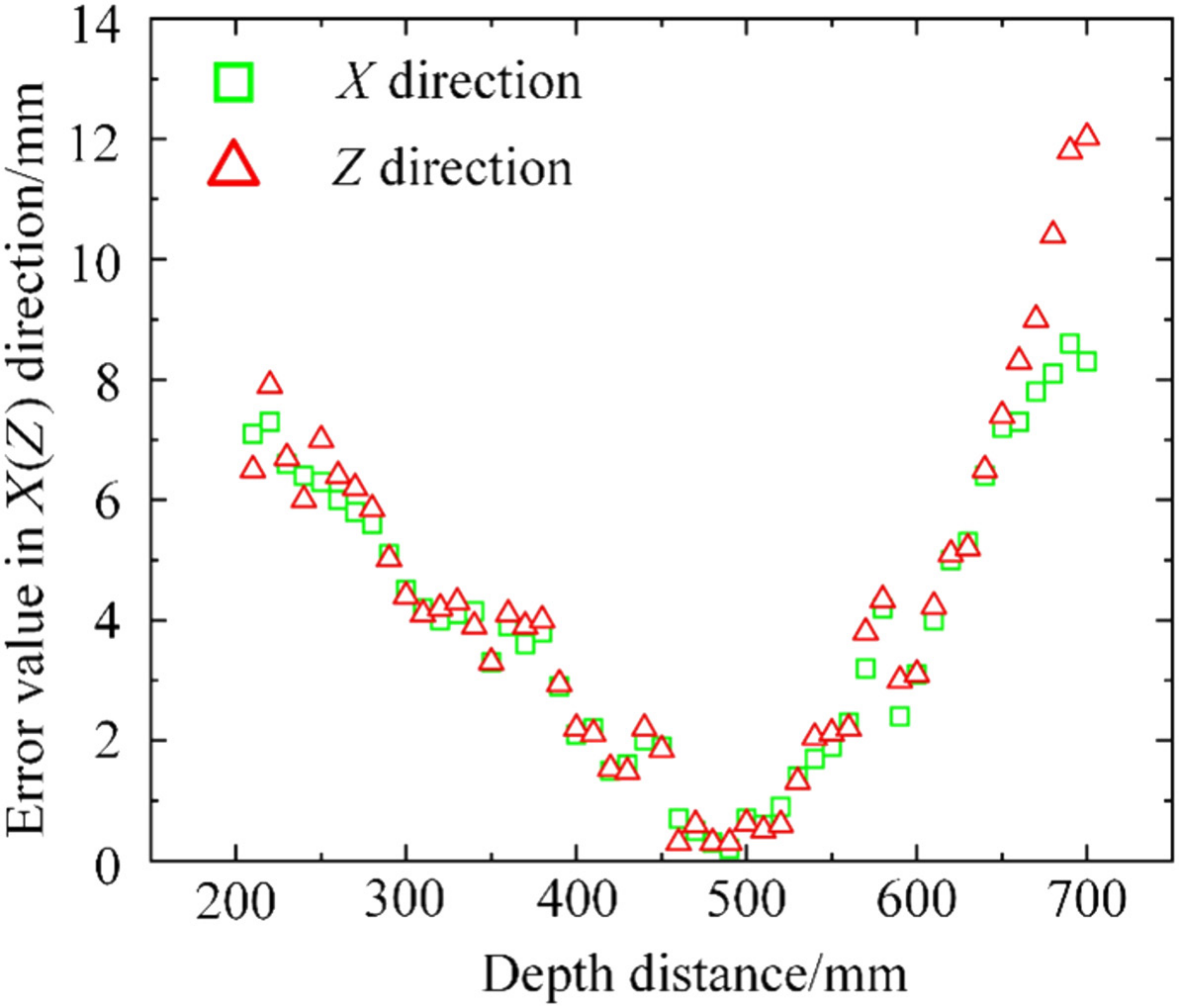
Schematic representation of the error distribution of picking points in the global coordinate system.

Taking a safflower image from a sunny day with light as an example, a depth-measurement test was conducted using the same target filament picking point at different depth distances. The depth distance between the center of gravity of the camera and filament picking point ranged from 200 to 700 mm. Fifty sets of depth-measurement errors are shown in Fig. 12 in the *X* and *Z* directions.

As shown in Fig. 12, the depth-measurement error of the camera continuously fluctuated with an increase in the depth distance between the depth camera and target filament. In particular, the depth-measurement error was within 13 mm in the *Z* direction. When the depth distance was 450 to 510 mm from the depth camera to the target filament, the depth-measurement error was minimized in the *Z* direction to less than 1 mm. When the depth distance exceeded 640 mm, the depth-measurement error was greater than 7 mm and continued to increase with increasing depth distance. The depth-measurement error was within 9 mm in the *X* direction. When the depth distance of the camera from the target filament was 450 to 510 mm, the depth-measurement error was minimized in the *X* direction. With an increase in the depth distance, the measurement error increased. Therefore, the optimal depth-measurement distance was 450 to 510 mm between the depth camera and target filament. The target visual-localization error between these distances was minimized to meet the harvesting-accuracy requirements.

## Picking-point real-time localization test

**Fig. 13.**
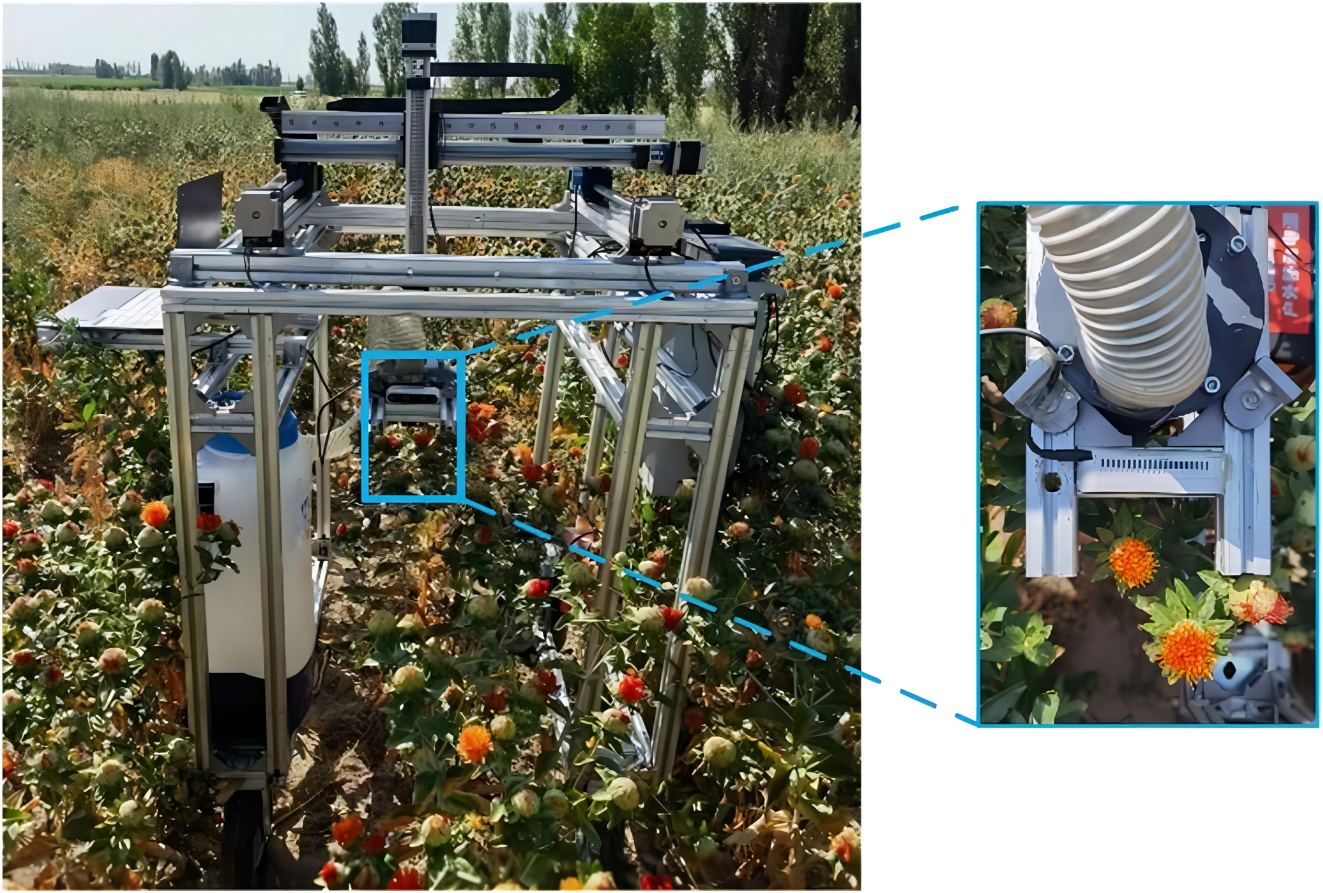
Validation tests and effects of safflower-harvesting robots.

The *XYZ* 3-axis sliding table module was used as the motion platform, the end effector was used as the picking robot, and a depth camera was used as the vision core, as shown in Fig. 13. The SDC-DeepLabv3+ algorithm detected real-time safflower scenes in the field under different weather conditions. Taking the correct rate, error rate and leakage rate of real-time frame detection as evaluation indexes, the real-time safflower detection results are shown in Table 5.

**Table 5.** Real-time detection results under different weather conditions

Types	No.	Frame detection correct rate/%	Frame detection error rate/%	Frame detection leakage rate/%
Sunny day with light	119	98.32	0	4.20
Sunny day with backlight	107	94.39	1.87	3.74
Overcast day with light	128	85.16	7.81	7.03
Cloudy day with light	115	88.70	5.22	6.09

Sunny day with light was applied to the safflower to improve the light intensity and brightness, which made the safflower color and texture features more obvious with no error rate. The correct rate of real-time frame detection is higher than the other cases by at least 3.93%, and the leakage rate is lower by at least 0.46%. Although the sunny day with backlight effect improves the light intensity and brightness on safflower, the filament itself blends with the complex surrounding environment, which is not conducive to identification and localization. Further analysis revealed that cloudy day with light was weak, making safflower color features unremarkable and with less relevant information. However, overcast day with light caused the color and texture features of the filaments to be blurred. The correct rate of real-time frame detection was the lowest, and the error and leakage rate were also relatively high.

The improved DeepLabv3+ was verified to localize the safflower-filament picking points under different weather conditions [4], as listed in Table 6. The results showed that the filament-localization and -picking success rates were the highest on sunny days with light, at 98.33% and 96.67%, respectively; the localization success rate was 92.50%. The filament leakage picking rate was also the lowest, at 3.33%. Furthermore, the color and texture features of the filament were blurred because of the influence of light and other shading conditions on cloudy days. The filament-localization and -picking success rates were lower than those under other weather conditions by at least 1.67% and 2.5%, respectively. The filament damage rate exceeded 0.74%.

**Table 6.** Field-harvesting test data for filaments

Types	No.	Localization success rate/%	Picking success rate/%	Filament leakage rate/%	Filament damage rate/%
Sunny day with light	120	98.33	96.67	3.33	7.07
Sunny day with backlight	120	95.00	92.50	7.50	6.01
Overcast day with light	120	87.50	85.83	14.17	7.81
Cloudy day with light	120	89.17	88.33	11.67	6.14

## Discussion

Accurate and rapid detection of safflower filaments in complex environments is of utmost importance for harvesting on schedule and preventing wilting [1,3]. Previous studies have demonstrated the identification and detection using deep learning methods combining background color and contour with top localization methods, which are prone to blindness and uncertainty in locating the filament picking point [60]. Previous studies have demonstrated improvements in algorithms that can alleviate the impact of complex backgrounds on the detection task [4,8,13]. However, these methods usually increase the model size and computational complexity, resulting in slower detection and causing severe filament damage. To solve the difficulty of accurately locating safflower-filament picking points with near-color backgrounds and irregular contours in complex environments, this study proposes a localization method for safflower filament picking-point detection using an improved DeepLabv3+ algorithm.

1. SDC-DeepLabv3+ uses a lightweight network, ShuffletNetV2, as the backbone network for detection. The background areas, contour edges, and interference with filament, fruit ball, and branch segmentation were reduced.

2. The dilated depth-separable convolution added 3 convolutional branches at different sampling rates. A CBAM was introduced to extract information on safflower features under different receptive fields.

3. The line segment that minimizes the distance between the barycenter coordinates and straight line was solved. The line was considered as the line position of the picking point and projected onto this line segment with the midpoint as the picking point.

The test results show that SDC-DeepLabv3+ enhanced the mIoU and mPA to 95.84% and 96.87%, respectively, compared with the original algorithm. This significantly improved the linkage of safflower filament features in the channel and space, suppressed background interference, and highlighted the filament more prominently. Furthermore, the FPS increased by 12.16 percentage points and the weights file size decreased by 180.78 MB. Compared with the other 5 segmentation algorithms, the SDC-DeepLabv3+ algorithm increased the mIoU and mPA by at least 1.71% and 1.23%, respectively [24–26,33]. The FPS increased by at least 9.11 f/s. In addition, the weights file size with only 25.54 MB were significantly smaller than those of the other models. A safflower filament picking-point test was performed under various weather conditions. The test results showed that the best distance was 450 to 510 mm, average localization success rate was 92.50%, and average picking success rate was 90.83%. This indicates that the performance index of the filament picking-point localization method improved significantly based on the proposed DeepLabv3+ algorithm, which exhibited a high performance stability and good adaptability.

In addition, the performance of the model on the test dataset is better than in training. The main reason for this is too much model regularization [61,62]. The difference between the model and the random classifiers during training is mainly due to too much Dropout, which causes the model to remove the set of random classifiers. The model at validation is large. However, Dropout will be automatically turned off during validation and all weak classifiers will be used, resulting in a relatively high test accuracy. Another reason is the lag of small batch statistics and the data preprocessing of the training set, such as geometric transformations (translation and rotation) with color transformations (contrast and brightness) and other operations [63,64]. Excessive preprocessing leads to changes in the distribution of the training set, making the test accuracy relatively high.

In this study, an SDC-DeepLabv3+ filament picking-point detection and localization method was developed by improving the algorithm in multiple ways to address the problem of near-color backgrounds and irregular contours of the picking point. The test verified that the algorithm had a good segmentation performance and obvious performance improvements over similar existing networks. However, extensive work can be carried out for in-depth research in the future:

1. This study only addressed safflower varieties grown in Xinjiang, China. Owing to the differences in the physical features related to the appearance of different safflower varieties, the robustness of the segmentation capability of the proposed algorithm must be verified. However, extending the application to localize more safflower varieties or even similar crops is necessary to obtain more comprehensive results.

2. The CBAM was introduced to improve the feature extraction of the improved algorithm. In future studies, the effects produced when CBAM is inserted at different locations in the backbone network should be considered. In addition, the channel or spatial attention mechanism should be considered at different positions in the algorithm. The extent of influence of the attention mechanism should be further derived from the segmentation performance of the improved algorithm.

## Data Availability

The image dataset used to support the findings of this study is available from the corresponding author upon request.
